# Exploring the role of in-patient magnetic resonance imaging use among admitted ischemic stroke patients in improving patient outcomes and reducing healthcare resource utilization

**DOI:** 10.3389/fneur.2024.1305514

**Published:** 2024-03-18

**Authors:** Mukesh Kumar, Steven Beyea, Sherry Hu, Noreen Kamal

**Affiliations:** ^**1**^Department of Industrial Engineering, Dalhousie University, Halifax, NS, Canada; ^2^Department of Diagnostic Radiology, Dalhousie University, Halifax, NS, Canada; ^3^IWK Health, Halifax, NS, Canada; ^4^Division of Neurology, Department of Medicine, Dalhousie University, Halifax, NS, Canada; ^5^Department of Community Health and Epidemiology, Dalhousie University, Halifax, NS, Canada

**Keywords:** MRI utilization, ischemic stroke, subacute stroke, stroke outcomes, length of stay, hospitalization cost

## Abstract

**Purpose:**

Despite the diagnostic and etiological significance of in-patient MRI in ischemic stroke (IS), its utilization is considered resource-intensive, expensive, and thus limiting feasibility and relevance. This study investigated the utilization of in-patient MRI for IS patients and its impact on patient and healthcare resource utilization outcomes.

**Methods:**

This retrospective registry-based study analyzed 1,956 IS patients admitted to Halifax’s QEII Health Centre between 2015 and 2019. Firstly, temporal trends of MRI and other neuroimaging utilization were evaluated. Secondly, we categorized the cohort into two groups (MRI vs. No MRI; in addition to a non-contrast CT) and investigated adjusted differences in patient outcomes at admission, discharge, and post-discharge using logistic regression. Additionally, we analyzed healthcare resource utilization using Poisson log-linear regression. Furthermore, patient outcomes significantly associated with MRI use underwent subgroup analysis for stroke severity (mild stroke including transient ischemic attack vs. moderate and severe stroke) and any acute stage treatment (thrombolytic or thrombectomy or both vs. no treatment) subgroups, while using an age and sex-adjusted logistic regression model.

**Results:**

MRI was used in 40.5% patients; non-contrast CT in 99.3%, CT angiogram in 61.8%, and CT perfusion in 50.3%. Higher MRI utilization was associated with male sex, younger age, mild stroke, wake-up stroke, and no thrombolytic or thrombectomy treatment. MRI use was independently associated with lower in-hospital mortality (adjusted OR, 0.23; 95% CI, 0.15–0.36), lower symptomatic neurological status changes (0.64; 0.43–0.94), higher home discharge (1.32; 1.07–1.63), good functional outcomes at discharge (mRS score 0–2) (1.38; 1.11–1.72), lower 30-day stroke re-admission rates (0.48; 0.26–0.89), shorter hospital stays (regression coefficient, 0.92; 95% CI, 0.90–0.94), and reduced direct costs of hospitalization (0.90; 0.89–0.91). Subgroup analysis revealed significantly positive association of MRI use with most patient outcomes in moderate and severe strokes subgroup and non-acutely treated subgroup. Conversely, outcomes in mild strokes (including TIAs) subgroup and acute treatment subgroup were comparable regardless of MRI use.

**Conclusion:**

A substantial proportion of admitted IS patients underwent MRI, and MRI use was associated with improved patient outcomes and reduced healthcare resource utilization. Considering the multifactorial nature of IS patient outcomes, further randomized controlled trials are suggested to investigate the role of increased MRI utilization in optimizing in-patient IS management.

## 1 Introduction

Stroke is a significant public health concern in Canada, ranking as the fifth leading cause of death and a major contributor to disability (1, 2). In the 2017–18 fiscal year, over 57,000 ischemic strokes (IS) and 41,000 transient ischemic attacks (TIA) were evaluated at Canadian emergency departments (ED) or hospitals, with a large proportion resulting in hospital admissions (3). The associated direct healthcare costs, both during hospitalization and post-discharge rehabilitation, are substantial.

Despite the excellent resolution and sensitivity provided by magnetic resonance imaging (MRI) (4), computed tomography (CT) remains the primary imaging modality during the first hour of patient arrival in the ED (hyper-acute phase), as recommended by IS guidelines (5). However, in the subacute phase, subsequent to decision for thrombolysis and endovascular thrombectomy (EVT) treatment, MRI holds considerable potential for providing diagnostic and etiological information, informing secondary prevention strategies (6, 7). Ideally, MRI would be performed within 24 h of patient arrival, after the primary CT imaging has been completed. However, there are observed disparities in the use of in-patient MRI for admitted IS patients, potentially attributed to logistic challenges for optimal MRI utilization in different geographic and institutional settings (8, 9). This suboptimal utilization has resulted in limited literature and a lack of clear guidelines regarding MRI use during the in-patient management of IS (5).

While studies conducted in insurance-based healthcare systems have linked in-patient MRI utilization to longer length of stay (LOS) and higher hospitalization costs (10, 11), there is a dearth of literature examining the utilization and outcomes of in-patient brain MRI for subacute IS patients in other care settings.

The current exploratory study aims to address gaps in knowledge by investigating the utilization of in-patient MRI for patients with IS beyond the hyper-acute stage and by evaluating the impact of MRI utilization on patient outcomes and healthcare resource usage at Queen Elizabeth II (QEII) Health Centre, the only comprehensive stroke center (CSC) in Nova Scotia, Canada.

## 2 Methods

### 2.1 Study databases and patient selection

The study adhered to the research policies and protocols established by Nova Scotia Health (NSH), the provincial health authority overseeing QEII Health Centre. Prior to initiation, it received approval from the Nova Scotia Health Research Ethics Board (File #1028219). QEII Health Centre operates as a level-1 trauma center with 800 beds, serving as the sole comprehensive stroke center in the province. The study period was from January 2015 to December 2019, excluding the COVID-19 pandemic period. The QEII Stroke Registry, containing comprehensive patient information, was used to identify IS or TIA patients admitted to the center. Data linkage with the Provincial Stroke Database provided patient age and re-admission details. To determine direct hospitalization costs and MRI timestamps, the study linked with NSH’s Affinity Decision Support database, validating MRI timestamps using the Secure Health Access Record.

Patient-level data from the aforementioned databases were utilized to identify adult patients diagnosed with IS or TIA. Patients with hemorrhagic stroke or stroke of unknown etiology were excluded from the analysis. Additionally, patients with a LOS exceeding 300 days were considered outliers and subsequently excluded. The included patients were categorized into two groups: the “MRI” group and the “No MRI” group, based on in-patient MRI acquisition. This categorization was independent of whether patients received single or multiple CT-based modalities at arrival. Almost all patients (99.3%) underwent a non-contrast CT scan, regardless of whether they subsequently underwent MRI or not. The study CSC utilizes a CT-based approach for acute stroke evaluation, wherein CT is obtained upon arrival for IS patients, and MRI is typically performed at a later stage if a need is perceived. A typical MRI protocol at our center includes T1 sagittal, T2 axial, T2-FLAIR axial, DWI and ADC, and GRE/SWI/SWAN sequences; however, additional sequences may be requested based on the requirements.

### 2.2 Study outcomes

For the first part of the study, we examined the utilization of neuroimaging modalities for in-patient IS over a five-year period (2015–2019). We assessed trends in MRI usage annually and compared it to other relevant techniques, including CT, CT angiography (CTA), and CT perfusion (CTP).

For the second part, we compared baseline characteristics and treatments provided in the ED, including tissue plasminogen activator (tPA) administration and EVT, between the “MRI group” and the “No MRI group.” In-hospital outcomes were evaluated, including mortality and symptomatic changes in neurological status. Symptomatic changes in neurological status were defined as any of the following: ischemia or intracranial/subarachnoid hemorrhage after TIA, multiple TIAs, hemorrhagic transformation after ischemia, ischemic recurrence or extension, and neurological deterioration of uncertain cause. Discharge and post-discharge outcomes measured home discharge disposition, good functional outcomes at discharge (mRS score 0–2), and re-admission rates for stroke within 30 days and 365 days after discharge. Finally, healthcare utilization outcomes, LOS and the direct cost of hospitalization, were analyzed. Direct costs encompassed expenses directly associated with patient care services, laboratory interventions, imaging exams, and management and administration expenses for each functional unit.

### 2.3 Statistical analysis

To study the annual trends of MRI and other neuroimaging utilization, we calculated the proportions of patients receiving specific neuroimaging tests stratified by year. Linear regression analysis was used to assess the significance of changes in neuroimaging utilization over time.

To compare baseline and ED treatment characteristics, descriptive statistics were used, and the Chi-square test and Mann–Whitney U test were applied for categorical and continuous variables, respectively. For binary patient outcomes, logistic regression was conducted as a multivariable regression analysis, while continuous healthcare resource utilization outcomes were analyzed using a Poisson log-linear model. The regression analyses were adjusted for age, sex, and stroke severity to isolate the independent effect of brain MRI on the outcomes. Stroke severity was assessed on arrival using a validated scale for stroke severity (12–14), which ranges from 1 to 10 (Supplementary Table S1). The higher scores indicated greater severity, categorized as mild (1–4), moderate (5–7), or severe (8–10). TIA was evaluated separately within this scoring system. Stroke severity was dichotomized as mild (including TIA) vs. moderate and severe strokes to facilitate regression analyses. Finally, patient outcomes significantly associated with MRI use in the previous step were subjected to subgroup analysis for stroke severity subgroups (mild stroke including TIA vs. moderate and severe stroke) and any acute stage treatment subgroups (tPA or EVT or both vs. no treatment). This subgroup analysis was conducted in conjunction with an age and sex-adjusted logistic regression model. All statistical analyses were conducted using IBM SPSS Statistics (Version 28.0.1.1, Armonk, NY), and a *p* value of less than 0.05 was considered statistically significant.

## 3 Results

A total of 2,251 adult stroke patients from the QEII Stroke Registry between 2015 and 2019 were initially identified. Among them, 277 had hemorrhagic strokes, and three had strokes of unknown cause, resulting in their exclusion from the analysis. Ten patients were excluded due to insufficient data in the linking databases, one patient under 18 years old was excluded, and four patients were considered outliers. The final analysis included 1956 patients who had experienced either an ischemic stroke or TIA as their index event.

The median age of patients was 73 years (interquartile range, IQR = 63–82), and 931 (47.6%) were female. Stroke severity distribution was as follows: 126 (6.4%) had TIA, 254 (13.0%) had mild strokes, 1,111 (56.8%) had moderate strokes, and 435 (22.2%) had severe strokes. While, the severity was undetermined for 32 (1.6%) patients.

### 3.1 Neuroimaging utilization

**Figure 1:**
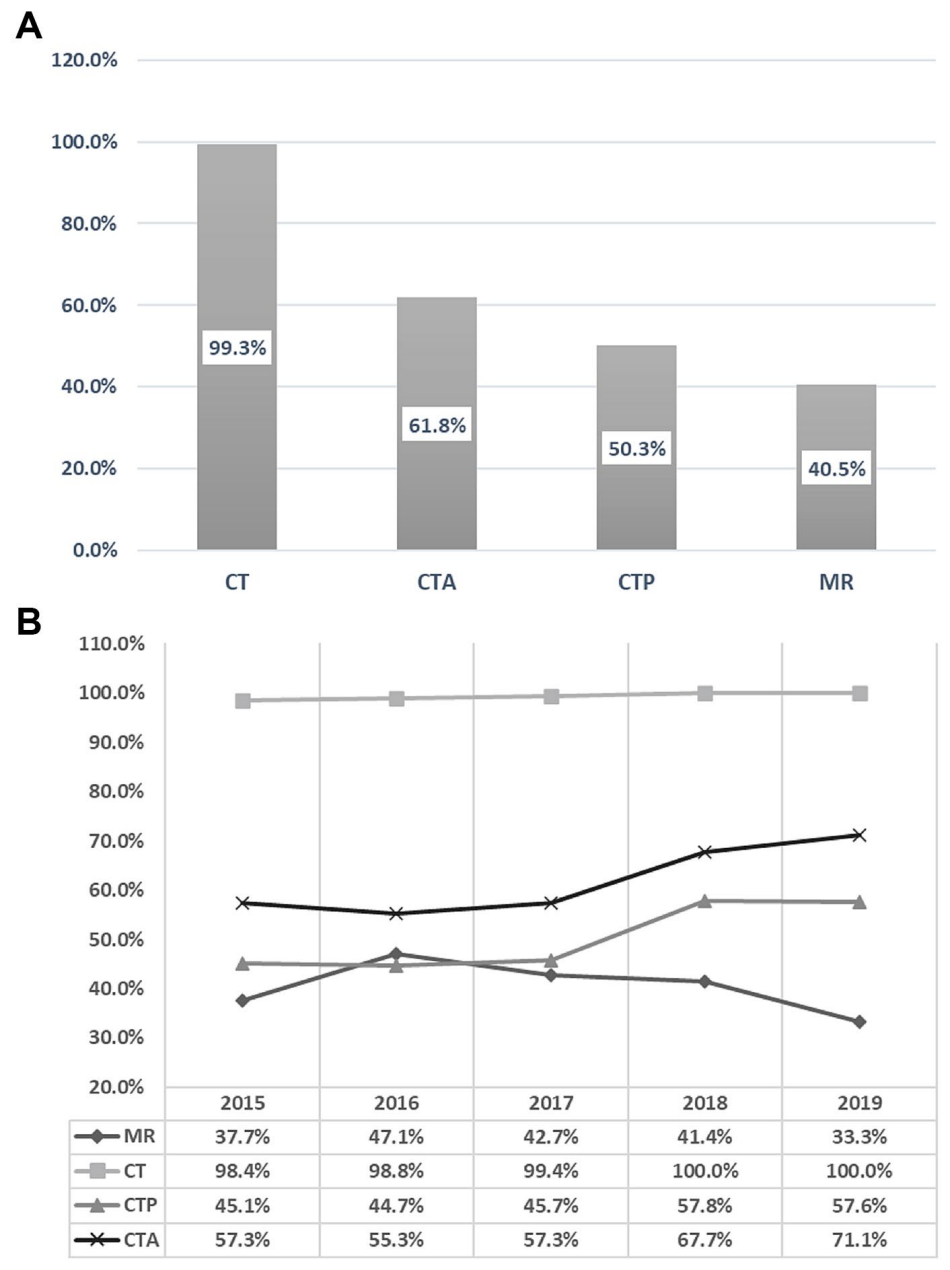
Overall 5-years utilization rates of various imaging modalities **(A)**. Annual trends of the various imaging modalities from 2015 to 2019 **(B)**.

During the five-year period, 793 patients (40.5%) underwent in-patient MRI, while CT was utilized by 1942 (99.3%), CTA by 1,209 (61.8%), and CTP by 984 (50.3%) patients. Between 2015 and 2019, there was a decrease of 4.4% in the absolute utilization of MRI, whereas the utilization rates of CT, CTA, and CTP increased by 1.6, 13.8, and 12.5%, respectively. Linear regression analysis showed a non-significant decrease in MRI utilization by −1.45% per year (95% confidence interval [CI] = −6.9 to 4.0, *p* = 0.459). Conversely, CT utilization significantly increased by 0.44% per year (95% CI = 0.2 to 0.6, *p* = 0.006), CTA utilization significantly increased by 4.00% per year (95% CI = 1.4 to 7.8, *p* = 0.046), and CTP utilization increased non-significantly by 3.81% per year (95% CI = −0.0 to 7.6, *p* = 0.051). The annual trends for all neuroimaging modalities are illustrated in Figure 1.

### 3.2 Baseline characteristics

For those who received an MRI, the median duration from symptom recognition to MRI was 56 h (IQR = 33–102). In the “MRI” group, a higher proportion of males (44.9% vs. 35.8%) and a lower median age (69 vs. 75 years) were observed compared to the “No MRI” group. The utilization rate of MRI was highest among patients with mild strokes (65.0%), followed by admitted TIA patients (47.6%), moderate strokes (44.4%), and severe strokes (15.4%). Additionally, patients arriving between 4.5 and 24 h from symptom recognition (55.7%) or beyond 24 h (55.2%) were more likely to undergo MRI than those arriving within 4.5 h (29.9%). Table 1 provides a comparison of baseline characteristics between patients who underwent MRI and those who did not.

**Table 1:** Comparison of the baseline characteristics and the emergency department management.

Characteristic	Missing cases, *N* (%)	MRI (*N* = 793)	No MRI (*N* = 1,163)	OR (95% CI)	*p*-value
Baseline characteristics					
Age in years, Median (IQR)	0 (0)	69 (58–77)	75 (66–84)		<0.001
Sex (Female), N (%)	0 (0)	333 (42.0)	598 (51.4)	0.68 (0.57–0.82)	<0.001
Severity of Stroke	32 (1.6)				<0.001
TIA, N (%)		60 (7.7)	66 (5.8)		
Mild stroke, N (%)		165 (21)	89 (7.8)		
Moderate stroke, N (%)		494 (62)	617 (54.1)		
Severe stroke, N (%)		67 (8.5)	368 (32.3)		
Wake-up stroke, N (%)	0 (0)	176 (22.1)	185 (15.9)	1.51 (1.20–1.90)	<0.001
EHS arrival, N (%)	0 (0)	394 (49.7)	835 (71.8)	0.39 (0.32–0.47)	<0.001
ASP activated, N (%)	0 (0)	233 (29.4)	639 (54.9)	0.34 (0.28–0.41)	<0.001
In-hospital stroke, N (%)	0 (0)	28 (3.5)	47 (4.0)	0.87 (0.54–1.40)	0.564
Symptom Recognition to Arrival Duration	0 (0)				<0.001
Within 4.5 h, N (%)		342 (43.1)	801 (68.9)		
4.5 h to 24 h, N (%)		234 (29.5)	186 (16.0)		
Beyond 24 h, N (%)		217 (27.4)	176 (17.1)		
Emergency department management					
tPA administered, N (%)	0 (0)	54 (6.8)	389 (33.4)	0.15 (0.11–0.20)	<0.001
EVT performed, N (%)	0 (0)	11 (1.4)	153 (13.2)	0.09 (0.05–0.17)	<0.001
Stroke unit admission, N (%)	0 (0)	745 (93.9)	1,082 (93.0)	1.16 (0.80–1.68)	0.425

The statistical comparisons were performed between categorical variables using the Chi-square test and between continuous variable (age) using the Mann–Whitney U test. TIA, transient ischemic attack; EHS, emergency health services; ASP, acute stroke protocol; tPA, tissue plasminogen activator; EVT, endovascular thrombectomy.

### 3.3 Emergency department treatment

Patients who received tPA had a significantly lower likelihood of undergoing MRI (tPA vs. no tPA, 12.2% vs. 48.8%). Similarly, patients who underwent EVT treatment had even lower odds of receiving MRI (EVT vs. no EVT, 6.7% vs. 43.6%). Table 1 provides a comparison of the ED treatments received by the “MRI” and “No MRI” groups.

### 3.4 Main study outcomes

Table 2 summarizes the unadjusted rates of all outcomes, and the age-, sex-, and stroke severity-adjusted odds ratios (OR) or regression coefficients with their corresponding 95% CI. The following sections provide an overview of the observed outcomes in study patients.

**Table 2:** Differences in in-hospital, discharge, post-discharge, and healthcare utilization outcomes between MRI and No MRI groups.

Outcome		Unadjusted incidence		Adjusted results	
In-hospital outcomes	Missing cases, N (%)	MRI (*N* = 793)	No MRI (*N* = 1,163)	Adjusted OR (95% CI)	*p-*value
In-hospital mortality, N (%)	0 (0)	26 (3.3)	193 (16.6)	0.23 (0.15–0.36)	< 0.001
Symptomatic neurological status change, N (%)	0 (0)	42 (5.3)	92 (7.9)	0.64 (0.43–0.94)	0.024
Discharge and post-discharge outcomes	Missing cases	MRI (N = 767)	No MRI (*N* = 970)	Adjusted OR (95% CI)	*P*-value
Home discharge, N (%)	0 (0)	461 (60.1)	498 (48.7)	1.32 (1.07–1.63)	0.009
Good functional outcomes (mRS 0–2), N (%)	9 (0.5)	386 (50.5)	333 (34.5)	1.38 (1.11–1.72)	0.004
Re-admission for stroke within 30 days of discharge, N (%)	0 (0)	17 (2.2)	37 (3.8)	0.48 (0.26–0.89)	0.020
Re-admission for stroke within 365 days of discharge, N (%)	0 (0)	60 (7.8)	72 (7.4)	1.05 (0.72–1.52)	0.806
Healthcare resource utilization outcomes	Missing cases	MRI (*N* = 793)	No MRI (*N* = 1,163)	Regression coefficient (95% CI)	*P*-value
Length of stay, Mean ± SD	0 (0)	14.88 ± 25.2	19.12 ± 33.1	0.92 (0.90–0.94)	< 0.001
Direct cost of hospitalization, $CAD Mean ± SD	15 (0.8)	$10,978 ± $19,622	$12,804 ± $27,072	0.90 (0.89–0.91)	< 0.001

The statistical comparisons were performed between categorical variables using the binary logistic regression and between continuous variables using the generalized linear regression with Poisson loglinear model. In the analysis of discharge and post-discharge outcomes, patients who died during their hospital stay were excluded.

#### 3.4.1 In-hospital outcomes

In the “No MRI” group, the unadjusted mortality rate was observed to be higher (16.6%) compared to the “MRI” group (3.3%), and following adjustments for covariates using binary logistic regression, MRI use was independently associated with lower mortality (adjusted OR, 0.23; 95% CI, 0.15–0.36; *p* < 0.001). Furthermore, the unadjusted rate of symptomatic changes in neurological status during hospitalization was lower among patients who underwent MRI (5.3%) compared to those who did not (7.9%), and these differences remained significant after adjustment (adjusted OR, 0.64; 95% CI, 0.43–0.94; *p* = 0.024).

#### 3.4.2 Discharge and post-discharge outcomes

For this analysis, patients who experienced in-hospital mortality were excluded, resulting in 767 patients included in the “MRI” group and 970 patients in the “No MRI” group. The “No MRI” group exhibited a significant association with home discharge disposition (adjusted OR, 0.1.32; 95% CI, 1.07–1.63; *p* = 0.009). Notably, good functional outcomes at discharge were independently associated with the use of MRI (adjusted OR, 1.38; 95% CI, 1.11–1.72; *p* = 0.004).

The adjusted odds of re-admission for stroke within 30 days of discharge were significantly higher among patients who did not receive MRI (adjusted OR, 0.48; 95% CI, 0.26–0.89; *p* = 0.020). However, the adjusted odds of re-admission for stroke within 1 year of discharge did not significantly differ between the “MRI” and “No MRI” groups (adjusted OR, 1.05; 95% CI, 0.72–1.52; *p* = 0.806).

**Figure 2:**
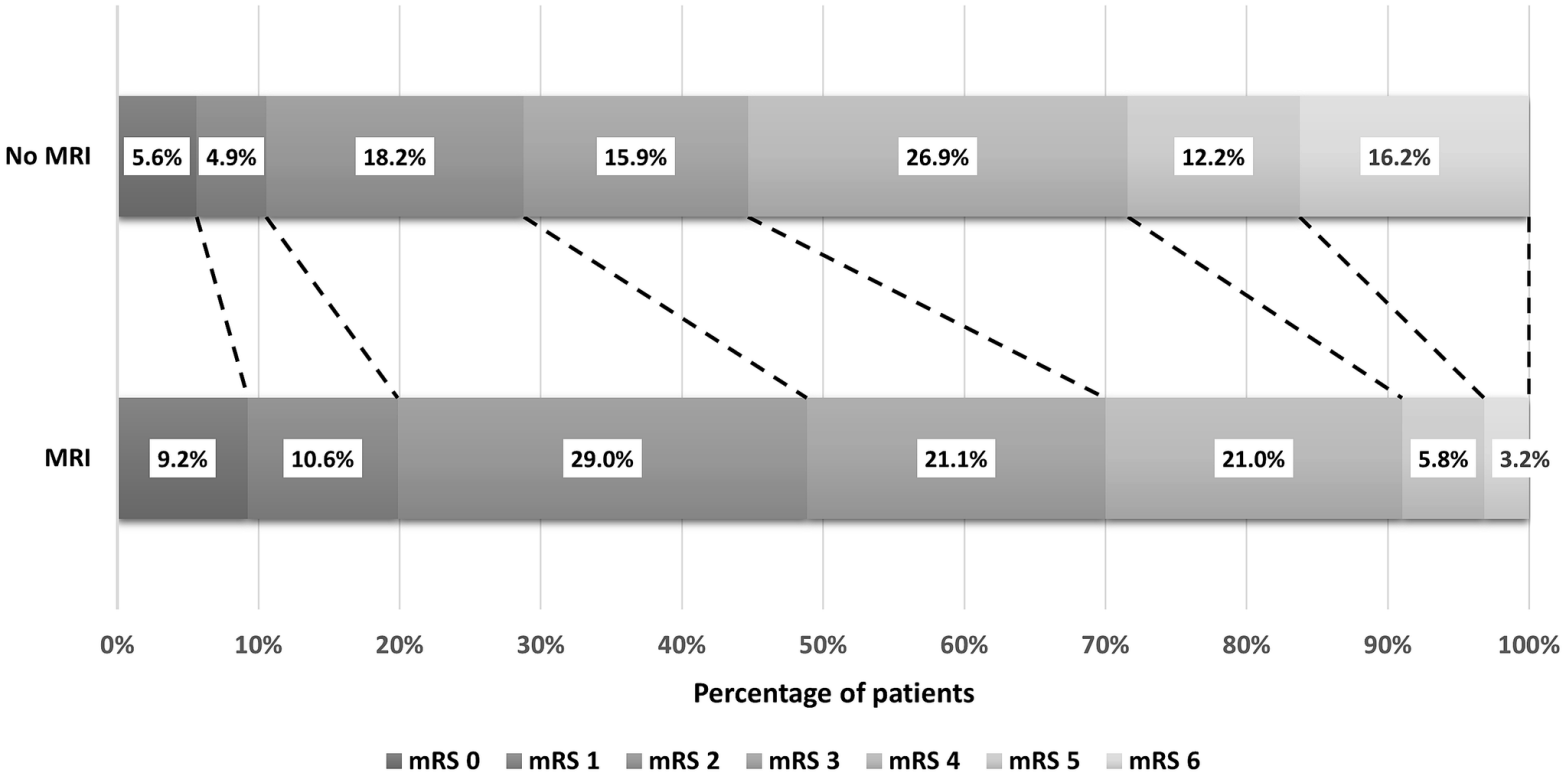
The distribution of mRS scores among ischemic stroke patients at discharge from the hospital.

Additionally, the distribution of unadjusted mRS scores at discharge was examined. In the non-MRI group, the highest proportion (26.9%) had an mRS score of 4, followed by scores of 2 (18.2%) and 6 (16.2%). Conversely, in the MRI group, the highest proportion (29.0%) had an mRS score of 2, followed by scores of 3 (21.1%) and 4 (21.0%). However, it is crucial to acknowledge that these rates are unadjusted and do not reflect the independent impact of MRI on functional outcomes. Figure 2 illustrates the distribution of mRS scores among IS patients using a stacked bar chart (Grotta bars).

#### 3.4.3 Healthcare resource utilization outcomes

Patients who did not undergo MRI had a longer unadjusted mean LOS (19.12 ± 33.1 days) compared to those who received MRI (14.88 ± 25.2 days), resulting in a mean LOS reduction of 4.2 days. The generalized linear regression analysis showed that MRI was independently associated with a decreased LOS (adjusted OR, 0.92; 95% CI, 0.90–0.94; *p* < 0.001). Furthermore, patients who did not undergo MRI had a higher mean direct cost of hospitalization (Canadian $12,804 ± 27,072 vs. Canadian $10,978 ± 19,622), and the mean cost reduction associated with MRI use was $1,826. The generalized linear regression analysis demonstrated that brain MRI was independently associated with a decrease in the direct cost of hospitalization (adjusted OR, 0.90; 95% CI, 0.89–0.91; *p* < 0.001).

#### 3.4.4 Subgroup analysis

As provided in Table 3, MRI use in the moderate and severe stroke subgroup was significantly associated with lower in-hospital mortality (adjusted OR, 0.22; 95% CI, 0.14–0.35; *p* < 0.001), lower symptomatic neurological status changes (adjusted OR, 0.57; 95% CI, 0.37–0.90; *p* = 0.015), higher home discharges (adjusted OR, 1.33; 95% CI, 1.06–1.66; *p* = 0.013), good functional outcomes at discharge (adjusted OR, 1.45; 95% CI, 1.13–1.84; *p* = 0.003), and lower 30-day stroke readmissions (adjusted OR, 0.39; 95% CI, 0.19–0.79; *p* = 0.010). Conversely, patient outcomes in the mild stroke (including TIA) subgroup were similar regardless of MRI use. Additionally, MRI usage in the subgroup with patients not acutely treated was significantly associated to lower in-hospital mortality (adjusted OR, 0.19; 95% CI, 0.12–0.31; *p* < 0.001), higher home discharges (adjusted OR, 1.57; 95% CI, 1.25–1.97; *p* < 0.001), and good functional outcomes at discharge (adjusted OR, 1.70; 95% CI, 1.35–2.15; *p* < 0.001), while outcomes in the acute treatment subgroup were comparable between MRI users and non-users.

**Table 3:** Subgroup analysis.

			Adjusted OR (95% CI)		
Outcome	Subgroup	Events/Total	No MRI	MRI	*p-*value
In-hospital outcomes					
In-hospital mortality	Stroke severity				
	TIA and mild stroke	4/380	1	0.82 (0.11–6.22)	0.847
	Moderate and severe stroke	214/1544	1	0.22 (0.14–0.35)	< 0.001
	Any acute treatment (tPA or EVT or both)				
	Yes	74/500	1	0.84 (0.31–2.25)	0.726
	No	145/1456	1	0.19 (0.12–0.31)	< 0.001
Symptomatic neurological status change	Stroke severity				
	TIA and mild stroke	24/380	1	1.13 (0.47–2.72)	0.793
	Moderate and severe stroke	109/1544	1	0.57 (0.37–0.90)	0.015
	Any acute treatment (tPA or EVT or both)				
	Yes	50/500	1	0.97 (0.36–2.61)	0.948
	No	84/1456	1	0.75 (0.48–1.19)	0.224
Discharge and post-discharge outcomes					
Home discharge	Stroke severity				
	TIA and mild stroke	318/376	1	1.19 (0.65–2.17)	0.573
	Moderate and severe stroke	606/1330	1	1.33 (1.06–1.66)	0.013
	Any acute treatment (tPA or EVT or both)				
	Yes	213/426	1	1.04 (0.57–1.92)	0.891
	No	720/1311	1	1.57 (1.25–1.97)	< 0.001
Good functional outcomes (mRS 0–2)	Stroke severity				
	TIA and mild stroke	300/375	1	1.08 (0.62–1.88)	0.793
	Moderate and severe stroke	416/1323	1	1.45 (1.13–1.84)	0.003
	Any acute treatment (tPA or EVT or both)				
	Yes	164/426	1	1.35 (0.72–2.53)	0.344
	No	555/1302	1	1.70 (1.35–2.15)	< 0.001
Re-admission for stroke within 30 days of discharge	Stroke severity				
	TIA and mild stroke	9/376	1	0.99 (0.21–4.65)	0.994
	Moderate and severe stroke	44/1330	1	0.39 (0.19–0.79)	0.010
	Any acute treatment (tPA or EVT or both)				
	Yes	25/426	1	0.24 (0.03–1.86)	0.170
	No	29/1311	1	0.90 (0.42–1.94)	0.795

The statistical comparisons were performed using the binary logistic regression. Stroke severity data were missing for 32 (1.6%) patients, and mRS data were missing for 9 (0.5%) patients. In the analysis of discharge and post-discharge outcomes, patients who died during their hospital stay were excluded. TIA, transient ischemic attack; tPA, tissue plasminogen activator; EVT, endovascular thrombectomy.

## 4 Discussion

The present study used the local stroke registry and relevant databases to investigate the trends in MRI utilization from 2015 to 2019 and its impact on clinical outcomes and healthcare resource utilization in patients hospitalized for IS. In the next sections, a comprehensive discussion of the study findings is presented.

### 4.1 MRI utilization

The study findings revealed that in-patient MRI utilization for IS patients was lower than other neuroimaging modalities, but still substantial. However, MRI usage trends varied across the study years, in contrast to previous US studies showing a consistent increase in MRI usage for IS patients over 10 years, ranging from 18 to 38% (15, 16). Furthermore, a comparative analysis between two academic urban hospitals in the US and Canada demonstrated significantly higher MRI utilization for admitted IS patients in the US hospital (95.7%) compared to the Canadian hospital (41.4%) (8). The Canadian hospital had more beds, physicians, discharge numbers, and occupancy rate, but the US center had six MRI scanners operating 24/7, while the Canadian center had only two MRI scanners operating 24 h on weekdays and 8 h on weekends. Limited MRI access likely contributes to the variations in utilization and may explain the inconsistent and declining trend observed in the current study. Additionally, the increased use of CTA and CTP scans for EVT treatments at our center might have stymied the expansion of MRI utilization.

### 4.2 Patient outcomes

This study’s most striking finding is the independent association between in-patient MRI utilization for IS and improved patient outcomes at various stages. While a direct causal relationship cannot be established, several potential reasons may explain these improved outcomes.

Firstly, MRI utilization in the ED provides significant benefits for accurate diagnosis, especially for suspected stroke mimics and specific stroke types like lacunar strokes and posterior circulation strokes (4, 17–22). This accurate diagnosis may reduce unnecessary hospital admissions, leading to shorter LOS and potential cost savings (23, 24). Moreover, these advantages may extend beyond the ED, positively impacting in-patient care and patient outcomes. Secondly, in-patient MRI use may have significant downstream effects on IS patient management, leading to improved outcomes. MRI may assist in the etiological work-up of IS patients by identifying characteristic infarct patterns, aiding in the detection of specific stroke etiologies like carotid stenosis (25), cardioembolism (26), and hypercoagulable conditions, including malignancies (27). This can guide further investigations, such as prolonged cardiac monitoring (26) or searching for underlying malignancies, leading to targeted treatment strategies like surgery or anticoagulation, thereby reducing recurrent stroke and disability. Our study’s findings align with previous research in insurance-based healthcare systems, which also demonstrated improved outcomes with in-patient MRI utilization for IS (11). Furthermore, MRI can be valuable for risk stratification in TIA and minor stroke patients, guiding aggressive treatments. In our study, 60% of such patients underwent MRI, and had significantly lower odds of in-hospital symptomatic neurological change and readmission within 30 days, suggesting reduced recurrent stroke risk. These findings align with post-hoc data from CHANCE (28) and POINT (29) studies, revealing increased recurrent IS risk within 90 days for MRI-DWI-positive minor stroke patients. Dual antiplatelet therapy was notably effective in preventing stroke recurrence in this group. Finally, MRI scans for moderate and severe stroke patients may be challenging, yet the derived information is valuable. MRI findings, such as hemorrhagic transformation on GRE or SWAN sequences, microbleed burden, or size of ischemic infarct on MRI-DWI, may guide anticoagulation timing to minimize hemorrhagic transformation (30).

Despite these arguments, the multifactorial nature of study outcomes is influenced by baseline group differences. It appears that older patients with more severe strokes who arrived early with an ASP activation had lower odds of undergoing MRI examination. This is likely due to our center typically not conducting follow-up MRI for tPA/EVT treated patients, who may be experiencing a stroke in progress leading to higher rates of poor short-term outcomes in the No MRI group. However, after adjusting for the most prominent covariates and conducting subgroup analysis, it was observed that the majority of benefits of using MRI were among those with moderate and severe strokes and those who were not treated with tPA/EVT. This finding contradicts the anecdotal belief that post-hyperacute MRI is only helpful among TIA or minor strokes.

### 4.3 Healthcare resources utilization

The existing scientific literature lacks comprehensive information regarding in-patient MRI use in IS. Our study, however, revealed that MRI utilization was associated with reduced healthcare resource usage, contradicting similar studies in insurance-based healthcare systems that reported higher resource utilization in MRI groups (10, 11, 15). The average cost of MRI in our study was $116, significantly lower than the unadjusted mean decrease of $1,826 in direct hospitalization costs associated with MRI utilization. This challenges the perception that combined use of CT and MRI is expensive and resource-intensive (31), suggesting that forgoing MRI based on these assumptions may impact patient outcomes. Furthermore, a study with an educational intervention found that reducing the combined use of CT and MRI did not significantly decrease overall charges for stroke patients receiving neurology services (32). Additionally, early MRI acquisition (within 12 h of arrival) in a study was linked to reduced length of stay for IS patients (33). Implementing in-patient MRI for IS within a universal healthcare system could result in cost-saving benefits, as supported by our current and previous research (34, 35).

### 4.4 Limitations and strengths

The present study has several limitations. First, its retrospective design introduces inherent differences between the study groups, particularly in the baseline characteristics. However, we mitigated most of these differences by using regression methods to adjust for confounding variables. Secondly, we acknowledge that mRS at discharge is not a reliable predictor of functional outcome compared to long-term mRS. However, post-discharge follow-up is not standard with our stroke registry, limiting our ability to analyze the impact of MRI use on long-term mRS. Furthermore, the study is based on a single-center registry, limiting the study population and generalizability. Moreover, the study center’s location in a small Canadian province may not represent the wider population of IS patients in Canada. Finally, the study duration from 2015 to 2019 may not reflect the latest data in the field, but it was chosen to avoid potential influences from the COVID-19 pandemic.

The study has notable strengths, such as comprehensive data on baseline characteristics, in-patient management, and post-discharge outcomes, allowing for adjusted regression analyses to ascertain the independent impact of MRI utilization on IS patient outcomes and healthcare resource usage.

## 5 Conclusion

This study provides evidence supporting the use of in-patient MRI in IS. The findings indicate that MRI utilization is associated with improved patient outcomes and lower healthcare resource utilization. In the subgroup analysis, the majority of benefits of using MRI were observed among those with moderate and severe strokes, strengthening the argument that post-hyperacute MRI is beneficial beyond TIA and minor stroke. However, given the multifactorial nature of IS patient outcomes, we advise caution in interpreting our findings and intend to position our results as an exploratory endeavor. To further support the idea of integrating MRI as a standard diagnostic tool in the management of hospitalized patients with IS, randomized controlled trials are needed to provide concrete evidence on the impact of MRI utilization on improved patient outcomes.

## Data availability statement

The datasets presented in this article are not readily available because of ethical and legal restrictions. Requests to access the datasets should be directed to the corresponding author.

## Ethics statement

The studies involving humans were approved by the Nova Scotia Health Research Ethics Board (File #1028219). The studies were conducted in accordance with the local legislation and institutional requirements. The ethics committee/institutional review board waived the requirement of written informed consent for participation from the participants or the participants’ legal guardians/next of kin because Informed written consent was waived due to impracticability criteria, primarily stemming from the non-identifiable data from a large sample size obtained retrospectively through a registry.

## Author contributions

MK: Conceptualization, Data curation, Formal analysis, Investigation, Methodology, Project administration, Software, Visualization, Writing – original draft, Writing – review & editing. SB: Conceptualization, Project administration, Supervision, Writing – review & editing. SH: Conceptualization, Project administration, Supervision, Writing – review & editing. NK: Conceptualization, Funding acquisition, Investigation, Methodology, Project administration, Resources, Supervision, Writing – review & editing.
