# Supplementary material for: Exploring the role of in-patient magnetic resonance imaging use among admitted ischemic stroke patients in improving patient outcomes and reducing healthcare resource utilization
Source: Front Neurol. 2024 Mar 18;15:1305514. doi: 10.3389/fneur.2024.1305514 (PMC10983768; doi:10.3389/fneur.2024.1305514)
Supplement: Supplementary file 1 [file Table_1.docx]

**Supplemental Table 1: Stroke Severity Scale used to assess neurological status upon arrival**

| Severity of stroke | Category | Observation |
| --- | --- | --- |
| **Mild** | 1 | neurological symptoms but no signs and no functional difficulties |
|  | 2 | neurological signs but no symptoms and no functional difficulties |
|  | 3 | neurological symptoms and signs but no functional difficulties |
|  | 4 | same as 3 but with reduced functional status |
| **Moderate** | 5 | impairment in ONE of the following domains: swallowing, self-care, ambulation, communication, comprehension |
|  | 6 | impairment in TWO domains |
|  | 7 | impairment in THREE domains |
| **Severe** | 8 | impairment in FOUR domains |
|  | 9 | impairment in FIVE domains |
|  | 10 | reduced consciousness |
| **TIA** |  | no symptoms or signs or functional difficulties at time of first assessment |
| "Impairment" refers to a loss of independence, requiring assistance from another person or a device. | | |
